# Whole Genome 5′-Methylcytosine Level Quantification in Cirrhotic HCV-Infected Egyptian Patients with and without Hepatocellular Carcinoma

**DOI:** 10.1155/2020/1769735

**Published:** 2020-10-02

**Authors:** Ahmed M. Awad, Wafaa S. Ragab, Nourhan Degheidy, Said Ahmed Ooda

**Affiliations:** ^1^Chemical Pathology Department, Medical Research Institute, Alexandria University, Egypt; ^2^Experimental and Clinical Internal Medicine Department, Medical Research Institute, Alexandria University, Egypt

## Abstract

DNA methylation is an epigenetic mechanism used by cells to control gene expression. DNA methylation is a commonly used epigenetic signaling tool that can hold genes in the “off” position. Chronic infection with hepatitis C virus (HCV) is considered a major risk for chronic liver impairment. It is the most common leading cause of HCC. The present work is aimed at studying whole genome 5′-methylcytosine levels in cirrhotic HCV-infected Egyptian patients. In the present study, 120 Egyptian adults were included. They were divided into two groups: group І (40 apparently healthy control subjects) and group ІІ (80 HCV-infected patients). Furthermore, group II was subdivided into 2 subgroups according to the presence of HCC in HCV-infected subjects. To all studied subjects, the level of 5-mC% was measured in peripheral blood. In the present study, the median of 5′-methylcytosine% in the control group (group I) was 2.5, in the HCV group (group IIa) was 2.45, and in the HCC group (group II b) was 2.25. A stepwise decrease in 5′-methylcytosine% from the control (group I) toward HCC (group IIb) was observed, taking into consideration that the stepwise global hypomethylation was not statistically significant (*p* = 0.811). There was a negative correlation between ALT and 5′-methylcytosine% (*p* = −0.029). From this study, we can conclude that global DNA 5′-methylcytosine% does not differ in HCV-infected cirrhotic patients and HCC patients when compared to normal controls. Consecutively, we had concluded that there is no impact of 5′-methylcytosine% on the development of liver cirrhosis or HCC. Moreover, the negative correlation between 5′-methylcytosine% and serum ALT level denotes a trend of decrease in 5′-methylcytosine% with more liver damage.

## 1. Introduction

Epigenetics refers to heritable changes in gene expression without changes to the underlying DNA sequence. Epigenetic changes are a regular and natural occurrence, but it can also be influenced by several factors including age, environmental factors, lifestyle, and disease state [1, 2]. Disordered epigenetic gene regulation is a feature of a number of important human diseases as neurodevelopmental disorders, cardiovascular disease, and cancer [3, 4]. A number of processes have been implicated in epigenetic gene regulation including DNA methylation, chromatin structure remodeling, histone modification, and noncoding RNAs [5].

DNA methylation refers to the addition of a methyl group (CH3) covalently to the base cytosine (C) in the dinucleotide 5′-CpG-3′ [6]. Genome-wide high-resolution DNA methylablast cell line demonstrated that 67.7% of CpGs are methylated [7]. The addition of methyl groups is controlled at several different levels in cells and is carried out by a family of enzymes called DNA methyltransferases (DNMTs) [8]. DNA methylation patterns are a product of the frequency of cytosine DNA methylation at specific sites along a strand of DNA [6]. DNA methylation occurs within the body of genes and between genes [9]. DNA methylation plays key roles in gene expression and regulation. It is an epigenetic signaling tool that locks genes in the “off position.” As in cancer cells, methylation within promoters serves to turn off critical genes (tumor suppressor genes) that could otherwise suppress tumorigenesis. It is also an important component in various cellular processes such as genomic imprinting, maintenance of chromosome stability, and X-chromosome inactivation [6]. DNA hypomethylation in cancer is as prevalent as cancer-linked hypermethylation [10]. So, the quantification of 5′-methylcytosine content or global methylation in diseased or environmentally impacted cells could provide useful information for detection of cancer [7]. Exposures to certain environmental factors can alter DNA methylation [11]. One of the questions asked while assessing the correlation between environmental exposure and epigenetic pattern is whether the pattern is a result of the exposure or it is a sign that the exposure has affected something else in the genome [12].

Hepatitis C virus (HCV) is common in the Middle East and in Africa [13]. Chronic hepatitis is known to induce changes in epigenetic machineries, including disruption of tissue and cell-specific DNA methylation patterns, resulting both in hyper- and hypomethylation of specific CPG sites [14]. These changes in turn may contribute to the exacerbation of chronic inflammation, thus producing a positive feedback loop between inflammatory and epigenetic changes which can promote proliferation and oncogenic transformation [15, 16]. HCV infection induces genome-wide, time-dependent changes in DNA methylation in infected mice with humanized livers [17]. Reactive oxygen species (ROS) induced by chronic HCV infection could change histone modification to the repressive form at CpG island-containing tumor suppressor gene promoters, leading to tumor suppressor gene inactivation. These findings suggest that chronic inflammation associated with HCV infection might cause epigenetic alterations by the immune response as well as through the induction of oxidative stress [17, 18, 19]. The precise molecular mechanism underlying HCV infection causing hepatocarcinogenesis is not fully understood. It has been proposed that HCV infection leads to hepatocarcinogenesis indirectly by viral-induced inflammation and oxidative stress. This microenvironment sets the stage for malignant transformation of hepatocytes through accumulation of both genetic and epigenetic changes [20].

To the best of our knowledge, there are no studies about the whole genome 5′-methylcytosine level in cirrhotic HCV-infected Egyptian patients which makes it noteworthy to quantitatively estimate the level of 5′-methylcytosine in the peripheral blood of the cirrhotic hepatitis C-infected Egyptian patients.

## 2. Materials and Methods

After approval of the Ethical Committee of the Medical Research Institute, Alexandria University, 120 subjects were included in the present study; patients were recruited from the Internal Medicine Department at Medical Research Institute Hospital. Informed consent was taken from all subjects who participated in the study. The subjects were divided into two groups:

(i) Group I (control group): it consisted of 40 apparently healthy volunteers of matched age and sex with the patient group
(ii) Group II (patient group): it consisted of 80 HCV-infected cirrhotic patients from the Internal Medicine Department at Medical Research Institute Hospital and was subdivided into 2 subgroups:
  - Group IIa (HCV group): 60 cirrhotic HCV-infected patients without HCC
  - Group IIb (HCC group): 20 cirrhotic HCV-infected patients with HCC

Exclusion criteria included any patient with liver disease not related to HCV or malignancies other than HCC.

To all the studied subjects, the following was done:

- Detailed history taken from all subjects including history of bleeding, encephalopathy, jaundice, and ascites
- Complete physical examination done to all subjects
- X-ray chest
- Abdominal ultrasound [21]: diagnosis of liver cirrhosis was based on clinical manifestations (jaundice, spider angiomata, palmar erythema, flappy tremors, ascites, and splenomegaly), biochemical markers (reversed ALT/AST ratio, hyperbilirubinemia, hypoalbuminemia, and increased INR), and ultrasonographic features (coarse parenchymal echogenicity with a nodular pattern, small-sized liver, hypertrophy of caudate lobe, ascites, splenomegaly, portal hypertension, and presence of portosystemic collaterals) of liver cirrhosis.

Moreover, Child-Pugh classification [22] was done for the patient group only; according to the Child-Pugh classification, patients with liver diseases were classified into 3 risk groups (A, B, and C) according to the numerical score obtained from Tables 1 and 2.

Diagnosis of hepatocellular carcinoma (HCC) in cirrhotic patients was based on multiphasic computerized tomography (CT) or dynamic contrast-enhanced magnetic resonance imaging (MRI). Diagnosis was based on the identification of the typical hallmarks of HCC which is the combination of hypervascularity in the late arterial phase (defined as arterial phase hyperenhancement (APHE) according to the Liver Imaging Reporting and Data System (LI-RADS) classification) and washout on portal venous and/or delayed phases, which reflects the vascular derangement occurring during hepatocarcinogenesis [23]. Staging of HCC was based on the Barcelona Clinic Liver Cancer (BCLC) staging system [24].

(v) Laboratory investigations: complete blood count [25] was assayed by the Sysmex xs-800i cell counter, and plasma prothrombin activity (PT) [26] was assayed by BFT ІІ Siemens, while INR was calculated by the following formula: INR = (PT patient/PT normal)^ISI^. Fasting serum glucose (FSG), albumin, total bilirubin, direct bilirubin, alanine aminotransferase (ALT), aspartate aminotransferase (AST), and gamma glutamyl transferase (GGT) [27] were analyzed on the Olympus AU400 Chemistry Autoanalyzer; serodetection of HCV IgG antibody and serodetection of hepatitis B by enzyme-linked immunosorbent assay (ELISA) technique (Murex Diagnostic limited, Dartford, England) [28, 29]; estimation of AFP by the chemiluminometric method on Immulite 1000 Siemens [30]; and HCV RNA real-time PCR [31] with the Mx3000P™ (Stratagene).

The molecular experiments that had been performed in the current study were whole genome 5′-methylcytosine level quantification [32] which included the following.

1. DNA extraction from peripheral blood leucocytes: DNA was purified from whole blood using the PureLink Genomic DNA kit for purification of genomic DNA; then, estimation of DNA concentration and purity had been done
2. Global DNA methylation determination kit: Methyl Flash™ Methylated DNA Quantification Kit (ELISA): the MethylFlash™ Methylated DNA Quantification Kit (ELISA) contains all reagents necessary for the quantification of global DNA methylation. In this assay, DNA is bound to strip wells that are specifically treated to have a high DNA affinity. The methylated fraction of DNA is detected using capture and detection antibodies and then quantified colorimetrically by reading the absorbance in a microplate spectrophotometer. The amount of methylated DNA is proportional to the OD intensity measured.

### 2.1. 5-mC Calculations

Simple calculation of the percentage of 5-mC in total DNA can be carried out using the following formula:

(1) $$\begin{array}{c} 5‐\text{mC}\%=\frac{\left(\text{sample OD}-\text{Negative control OD}\right)÷\text{input sample DNA in ng }}{\left(\text{Positive control OD}-\text{Negative control OD}\right)\times 2*÷5\text{ng}}\times 100\%. \end{array}$$

### 2.2. Statistical Analysis [33]

Data analysis was performed by using SPSS for Windows, version 20 (Statistical Package for the Social Sciences, Chicago, USA).

## 3. Results

### 3.1. 5′-Methylcytosine% (Tables 3–7 and Figures 1 and 2)

Table 3 shows that there is no statistically significant difference between the control group (group I) (median = 2.5) and the patient group (group ІІ) (median = 2.39) as regards 5′-methylcytosine% (*p* = 0.526).

**Figure 1:**
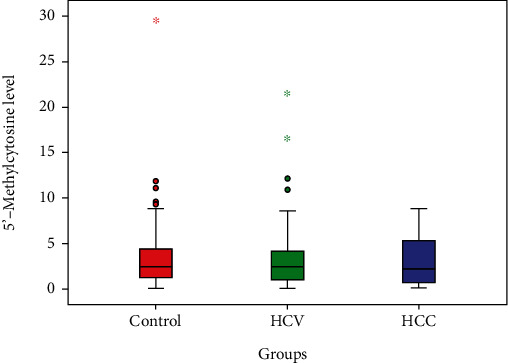
Statistical analysis between studied groups as regards 5′-methylcytosine% level.

Table 4 and Figure 1 show that there is no statistically significant difference between studied groups as regards 5′-methylcytosine% (*p* = 0.811). But there was a stepwise decrease in 5′-methylcytosine%, where in the control group, its median was 2.5; in the HCV group, its median was 2.45; and in the HCC group, its median was 2.25.

**Figure 2:**
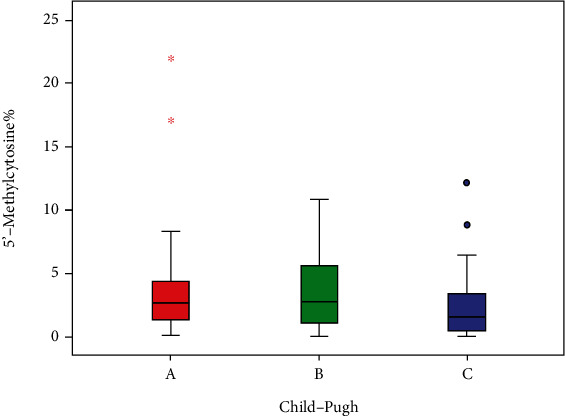
Statistical analysis of 5′-methylcytosine% in HCV patients with different Child-Pugh classes.

Table 5 and Figure 2 show that when HCV patients (group IIa) were subdivided according to their Child-Pugh class, there was no statistically significant difference between different classes as regards 5′-methylcytosine% (*p* = 0.443).

Tables 6 and 7 show that 5′-Methylcytosine% had no statistically significant impact on development of either liver cirrhosis or HCC, as the odds ratio = 0.967 (0.887-1.054), *p* = 0.442, and 1.032 (0.892-1.194), *p* = 0.674, respectively.

There was no statistically significant correlation between 5′-methylcytosine% and any of the studied parameters such as age, AFP, HCV viral load, or Child-Pugh class with the exception of the serum ALT level.

**Figure 3:**
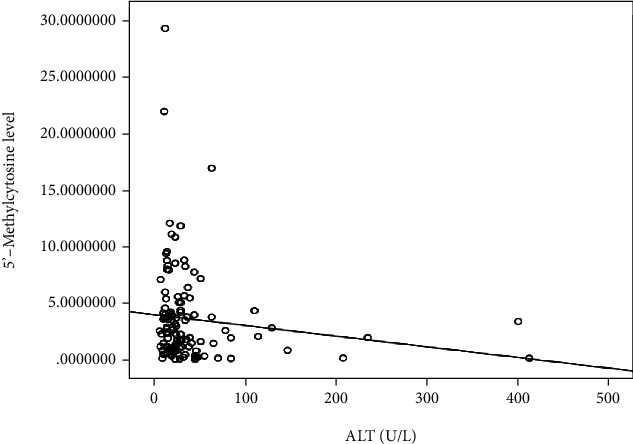
Scatter graph shows a correlation between 5′-methylcytosine and ALT.

Figure 3 shows that there was a negative significant correlation between serum ALT level and 5′-methylcytosine% in the studied groups (*p* = 0.029).

## 4. Discussion

Almost 20-30% of the patients with persistent HCV infection develop liver cirrhosis within 20-30 years after infection, while the estimated incidence of HCC in those patients is 1-8% per year [34]. Thus, virus-associated hepatocarcinogenesis persists to be a serious matter, and an explanation of the mechanism of virus-associated carcinogenesis in the liver is urgently needed [35]. Epigenetic changes including DNA methylation and histone modifications as well as genetic aberrations are accumulated in cancer cells [36]. DNA methylation is a common epigenetic mechanism that controls gene expression into the eukaryotic cells. DNA methylation acts as an epigenetic signaling tool that can hold genes in the “off” position [37]. Most CpG dinucleotides in the human genome are methylated. However, unmethylated CpGs are not distributed randomly but are clustered together in “CpG islands,” which are in the promoter region of many genes (the region that facilitates transcription of a particular gene) [38]. The global distribution of methylation in mammals has placed a challenge to researchers in terms of finding out whether methylation is a default state or is targeted at specific gene sequences. However, CpG islands are generally found in close proximity to transcription start sites, suggesting that there is an established recognition system [39]. A relation between chronic inflammation and DNA methylation has been suggested for a long time; however, the key factors linking them are still not completely understood [16].

Chronic liver inflammation is known to induce changes in epigenetic machineries, including disruption of tissue- and cell-specific DNA methylation patterns, resulting in both hyper- and hypomethylation of specific CPG sites [14]. The present work is aimed at studying whole genome 5′-methylcytosine levels in cirrhotic hepatitis C-infected Egyptian patients.

In the present study, 120 Egyptian adults were included. They were divided into two groups: group І (40 apparently healthy control subjects) and group ІІ (80 HCV-infected patients from the Internal Medicine Department of Medical Research Institute Hospital, Alexandria University). Group ІІ was further subdivided into two subgroups: group ІIa (60 HCV-infected cirrhotic patients without HCC) and group ІІb (20 HCV-infected cirrhotic patients with HCC). In the present study, there was no statistically significant difference between the studied groups according to age as all the studied groups were of comparable age. There was no significant correlation found between age and 5′-methylcytosine% (*p* = 0.356).

In contrast with our study, Gomes et al. [40] stated that there was global DNA hypomethylation in the peripheral blood sample which was measured in 126 old-aged individuals by using a high-throughput ELISA-based method. In the present study, the median of 5′-methylcytosine% in the control group was 2.5%, in group IIa 2.45%, and in group IIb 2.25%. A stepwise decrease in 5′-methylcytosine% from the control group toward group IIb was observed, but taking into consideration that this stepwise global hypomethylation was not statistically significant (*p* = 0.811). The forementioned finding is in agreement with the study of Wijetunga et al. [41] who isolated DNA from peripheral lymphocytes of 10 chronically HCV-infected patients and 10 HCV-negative controls. The global DNA methylation profiles of HCV-infected patients were not obviously different to those of uninfected patients (*p* > 0.05), and it was supposed that these results do not support that the chronic inflammatory process itself induces DNA methylation changes as a generalized effect in the body. Also, the current study is in agreement with another study done by Okamoto et al. (2014) [17] who assessed genome-wide DNA methylation status in humanized liver mice and found no reproducible change of the DNA methylation amount in HCV-infected cells in comparison with the uninfected cells (*p* = 0.783). Okamoto et al. study data demonstrated that in vitro HCV infection itself did not induce DNA methylation changes.

In contrast to our study, Nishida et al. [42] concluded that certain DNA methylation changes include global hypomethylation which already occurred in liver cirrhosis caused by HCV infection and persisted in HCC, where the mean methylation level% in normal liver tissue was 0.72, in cirrhotic liver tissue was 0.69, and in cancerous liver tissue was 0.35 (*p* < 0.001). Also, several studies have shown that HCV protein could induce regional hypermethylation of specific tumor suppressor genes and it could be an important player in hepatitis virus-induced epigenetic aberrations [43, 44]. Also, DNA methylation changes with HCV viral infection were reported to be time-dependent and progresses slowly, and that global DNA hypomethylation could be one from epigenetic changes that occur from HCV viral proteins [41].

In the present study, there was more decrease in the 5′-methylcytosine% level (although it is not significant) in HCV patients suffering from more deterioration in liver function and higher degree of liver cirrhosis. When 5′-methylcytosine% levels were compared among the studied HCV-infected cirrhotic patients (group IIb) after classifying them according to Child-Pugh classes, it was found that the median of 5′-methylcytosine% in Child A patients was 3.85%, in Child B patients was (2.35%), and in Child C patients was (2.26%) (*p* = 0.443).

In the present study, we found a negative correlation between ALT and 5′-methylcytosine% (*p* = 0.029). In agreement with our study, Wu et al. [45] found a negative significant correlation between ALT and methylation levels. Chen et al. [46] noted that HCC patients with a tumor size ≥ 5 cm showed a lower 5′-methylcytosine% content and a higher level of serum ALT. From these findings, it could be speculated that more liver damage is associated with a high ALT level and global DNA hypomethylation.

In the current study, we found by univariate logistic regression analysis that 5′-methylcytosine% is not a risk factor for liver cirrhosis development, as the odds ratio was 0.967 (0.887-1.054) and *p* = 0.442. Also, 5′-methylcytosine% did not have a statistically significant impact on the development of HCC where the odds ratio was 1.032 (0.892-1.194) and *p* = 0.674. In addition, there was no correlation between 5′-methylcytosine% and alpha fetoprotein *p* = 0.750. In agreement with our results, Chen et al. (2013) [46] quantified 5′-methylcytosine% in genomic DNA from HCC tumor tissue and relevant tumor adjacent normal tissues, where the mean contents of 5′-methylcytosine% in genomic DNA from matched pair tumor tissues and tumor adjacent tissues were 0.67% and 0.84%, respectively, and they concluded that hypomethylation (decreased 5′-methylcytosine%) is not associated with liver cirrhosis stages (*p* = 0.567), liver inflammation stages (*p* = 0.739), or tumor stages (*p* = 0.68). In contrast to our study, Di et al. [47] reported that the median of 5′-methylcytosine% in HCC cases is significantly lower than that in the control group, and they concluded that the decrease in 5′-methylcytosine% has a statistically significant impact on the development of HCC, odds ratio = −1.9 (1.4-2.6), *p* ≤ 0.001. Also, Umer et al. [43] suggested that global DNA hypomethylation may be useful as a biomarker of HCC susceptibility. Also, Michailidi et al. [48] extracted DNA from frozen primary HCC and normal liver (noncirrhotic tissue obtained from autopsies) tissue samples and observed that there was statistically significant genome-wide hypomethylation in the tumor tissues when compared to normal samples.

## 5. Conclusions and Recommendations

From this study, we can conclude that global DNA 5′-methylcytosine% does not differ in HCV-infected cirrhotic patients and HCC patients compared with normal controls, denoting that the chronic HCV inflammatory process itself could not influence the global DNA methylation status and there was any correlation between age, AFP, HCV viral load, or Child-Pugh class in the studied patients and 5′-methylcytosine%; consecutively, we had concluded that there was no impact of 5′-methylcytosine% on development of liver cirrhosis or HCC.

Moreover, it had been concluded from the current study that 5′-methylcytosine% is negatively correlated with the serum ALT level, denoting a trend of decrease in 5′-methylcytosine% with more liver damage.

As the sample size may be a limitation of our study, it is recommended to reconduct the same study on a larger sample size at the peripheral blood and tissue level to minimize the probability of false-negative or false-positive results.

## Figures and Tables

**Table 1:** Child-Pugh scoring.

	1	2	3
Encephalopathy	None	Grades 1-2	Grades 3-4
Ascites	None	Mild/moderate	Severe
Serum albumin (g/dl)	>3.5	2.8-3.5	<2.8
Serum total bilirubin (mg/dl)	<2	2-3	>3
INR	<1.7	1.7-2.3	>2.3

**Table 2:** Child-Pugh classification.

Risk group	Numerical score
(1) Child-Pugh class A	5-6 points
(2) Child-Pugh class B	7-9 points
(3) Child-Pugh class C	10-15 points

**Table 3:** Statistical analysis between groups I and II as regards 5′-methylcytosine%.

	Control (*N* = 40)	Cases (*N* = 80)	Test of significance (*p*)
5′-Methylcytosine% median (Min–Max)	2.50 (0.079-29.36)	2.39 (0.082-22)	(*U* = 1486, *p* = 0.526)

*U*: Mann-Whitney test.

**Table 4:** Statistical analysis between studied groups as regards 5′-methylcytosine%.

	Control (*N* = 40)	HCV (*N* = 60)	HCC (*N* = 20)	Test of significance (*p*)
5′-Methylcytosine% median (Min–Max)	2.50 (0.079-29.36)	2.45 (0.08-22)	2.25 (0.16-8.86)	(*H* = 0.420, *p* = 0.811)

*H*: Kruskal-Wallis test.

**Table 5:** Statistical analysis between different Child classes in group IIa patients as regards 5′-methylcytosine level%.

	Child class A (*N* = 16)	Child class B (*N* = 34)	Child class C (*N* = 30)	Test of significance (*p*)
5′-Methylcytosine% median (Min–Max)	3.85 (0.08-17)	2.35 (0.13-22)	2.26 (0.19-8.87)	(*H* = 1.630, *p* = 0.443)

*H*: Kruskal-Wallis test.

**Table 6:** Odds ratio between the control and patient groups.

	Control group І		Case group ІІ		OR^1^ (95% CI^2^)	*p*
	No.	%	No.	%		
5′-Methylcytosine%	40	33.3	80	66.7	0.967 (0.887-1.054)	0.442

^1^OR: odds ratio; ^2^CI: confidence interval.

**Table 7:** Odds ratio between the HCV group and the HCC group.

Parameter	HCV group ІІa		HCC group ІІb		OR^1^ (95% CI^2^)	*p*
	No.	%	No.	%		
5′-Methylcytosine%	20	25	60	75	1.032 (0.892-1.194)	0.674

^1^OR: odds ratio; ^2^CI: confidence interval.

## Data Availability

Research data is provided in the supplementary file, and any other data will be provided whenever requested. For clinical and lab data for individual cases, refer to the supplementary file.

## Supplementary Materials

Supplementary Materials Clinical and laboratory data.
