# Supplementary material for: Whole Genome 5′-Methylcytosine Level Quantification in Cirrhotic HCV-Infected Egyptian Patients with and without Hepatocellular Carcinoma
Source: Int J Genomics. 2020 Oct 2;2020:1769735. doi: 10.1155/2020/1769735 (PMC7556053; doi:10.1155/2020/1769735)
Supplement: Supplementary Materials — Clinical and laboratory data. [file 1769735.f1.doc]

**Supplementary clinical and lab data for individual case**

Table Іa: Clinical findings of control group І

| **No** | **Age (years)** | **sex** | **Hb(g/dl)** | **WBCs x1000** | **Plts x1000** | **PT(sec)** | **INR** |
| --- | --- | --- | --- | --- | --- | --- | --- |
| **1** | 58 | F | 13 | 5 | 250 | 12.5 | 1.1 |
| **2** | 58 | M | 14 | 6 | 300 | 12 | 1 |
| **3** | 58 | F | 13 | 6.25 | 350 | 12 | 1 |
| **4** | 58 | F | 12 | 4.25 | 250 | 12 | 1 |
| **5** | 58 | F | 13 | 5.5 | 200 | 12.1 | 1 |
| **6** | 58 | F | 13 | 5 | 230 | 12.5 | 1.1 |
| **7** | 56 | F | 12.5 | 5 | 250 | 13 | 1.2 |
| **8** | 50 | F | 13 | 7 | 300 | 13.1 | 1.2 |
| **9** | 55 | M | 14.5 | 8.32 | 250 | 12.1 | 1 |
| **10** | 56 | F | 13 | 10 | 300 | 11.5 | 1 |
| **11** | 58 | M | 14 | 5.65 | 200 | 12 | 1 |
| **12** | 58 | F | 14 | 5 | 200 | 12.1 | 1 |
| **13** | 55 | M | 13 | 5.25 | 200 | 12 | 1 |
| **14** | 58 | F | 14 | 8 | 190 | 11.5 | 1 |
| **15** | 58 | M | 15 | 7.23 | 200 | 11.5 | 1 |
| **16** | 58 | F | 14 | 5 | 200 | 13.5 | 1.3 |
| **17** | 55 | F | 14 | 6 | 300 | 13 | 1.2 |
| **18** | 45 | F | 14 | 6.15 | 200 | 13 | 1.2 |
| **19** | 55 | M | 16 | 10 | 350 | 12 | 1.2 |
| **20** | 19 | F | 12 | 8.5 | 170 | 11.9 | 1 |
| **21** | 58 | M | 15 | 9 | 200 | 12 | 1 |
| **22** | 50 | F | 12 | 6.32 | 400 | 11 | 1 |
| **23** | 40 | M | 14 | 7 | 200 | 12 | 1 |
| **24** | 55 | F | 12 | 6.45 | 150 | 11.5 | 1 |
| **25** | 57 | F | 13 | 5 | 250 | 11.7 | 1 |
| **26** | 58 | F | 12 | 6.91 | 200 | 12 | 1 |
| **27** | 58 | M | 14 | 8.14 | 300 | 13 | 1.2 |
| **28** | 55 | F | 12 | 9 | 250 | 12 | 1 |
| **29** | 30 | M | 15 | 8.32 | 350 | 13.5 | 1.3 |
| **30** | 55 | M | 14 | 7.14 | 400 | 12 | 1 |
| **31** | 58 | F | 13 | 5.32 | 300 | 12 | 1 |
| **32** | 57 | M | 15 | 4.5 | 250 | 11.2 | 1 |
| **33** | 55 | M | 14.5 | 5 | 300 | 12 | 1 |
| **34** | 58 | M | 15 | 5 | 350 | 12 | 1 |
| **35** | 57 | M | 16 | 8.32 | 200 | 11.9 | 1 |
| **36** | 55 | F | 12 | 5.5 | 190 | 12 | 1 |
| **37** | 58 | M | 14 | 4.25 | 200 | 11 | 1 |
| **38** | 58 | F | 13 | 5 | 350 | 12 | 1 |
| **39** | 58 | F | 11.5 | 7.15 | 250 | 12 | 1 |
| **40** | 55 | M | 14 | 4.25 | 300 | 12 | 1 |
| **median** | 57 |  | 14 | 6 | 250 | 12 | 1 |
| **(min-max)** | 19-58 |  | 12-16 | 4-10 | 150-400 | 11-13.5 | 1-1.3 |
| **Refrence range** |  |  | F:11.7-15.5  M:12.5-17.5 | 4-11 | 150-450 | 11.5-14 | To 1.3 |

Table Іb: continued clinical findings of control group:

| **No** | **Glucose**  **(mmol/l)** | **Creatinine**  **(umol/l)** | **Albumin**  **(g/l)** | **T.bil**  **(umol/l)** | **D.bil**  **(umol/l)** | **GGT**  **(U/L)** | **AST**  **(U/L)** | **ALT**  **(U/L)** |
| --- | --- | --- | --- | --- | --- | --- | --- | --- |
| **1** | 4.7175 | 70.72 | 42 | 5.13 | 1.71 | 16 | 20 | 11 |
| **2** | 5.217 | 70.72 | 45 | 3.42 | 1.71 | 15 | 20 | 14 |
| **3** | 4.995 | 70.72 | 44 | 6.84 | 1.71 | 15 | 26 | 30 |
| **4** | 4.7175 | 79.56 | 48 | 3.42 | 1.71 | 12 | 16 | 19 |
| **5** | 5.106 | 44.2 | 49 | 5.13 | 1.71 | 10 | 15 | 11 |
| **6** | 5.2725 | 70.72 | 45 | 8.55 | 3.42 | 24 | 17 | 15 |
| **7** | 5.0505 | 70.72 | 45 | 10.26 | 3.42 | 23 | 15 | 13 |
| **8** | 4.995 | 61.88 | 45 | 8.55 | 3.42 | 13 | 20 | 15 |
| **9** | 5.2725 | 88.4 | 40 | 15.39 | 3.42 | 20 | 25 | 30 |
| **10** | 4.995 | 97.24 | 45 | 15.39 | 6.84 | 25 | 34 | 20 |
| **11** | 4.44 | 44.2 | 55 | 10.26 | 1.71 | 30 | 19 | 20 |
| **12** | 4.995 | 61.88 | 40 | 11.97 | 3.42 | 25 | 30 | 33 |
| **13** | 5.2725 | 79.56 | 43.2 | 11.97 | 3.42 | 10 | 14 | 8 |
| **14** | 4.995 | 70.72 | 40 | 8.55 | 1.71 | 15 | 20 | 25 |
| **15** | 4.884 | 61.88 | 41 | 8.55 | 3.42 | 20 | 22 | 24 |
| **16** | 4.7175 | 61.88 | 45 | 8.55 | 3.42 | 15 | 15 | 14 |
| **17** | 4.44 | 61.88 | 45 | 8.55 | 3.42 | 15 | 22 | 20 |
| **18** | 4.995 | 70.72 | 40 | 8.55 | 3.42 | 40 | 30 | 35 |
| **19** | 4.44 | 70.72 | 40 | 8.55 | 3.42 | 15 | 25 | 15 |
| **20** | 5.2725 | 79.56 | 45 | 8.55 | 3.42 | 15 | 20 | 15 |
| **21** | 5.1615 | 70.72 | 45 | 8.55 | 1.71 | 15 | 19 | 9 |
| **22** | 5.2725 | 97.24 | 45 | 5.13 | 1.71 | 15 | 24 | 25 |
| **23** | 4.995 | 61.88 | 45 | 8.55 | 3.42 | 15 | 21 | 16 |
| **24** | 4.7175 | 88.4 | 40 | 8.55 | 5.13 | 15 | 22 | 20 |
| **25** | 4.995 | 88.4 | 50 | 15.39 | 5.13 | 25 | 35 | 34 |
| **26** | 5.2725 | 79.56 | 40 | 13.68 | 6.84 | 30 | 30 | 12 |
| **27** | 4.995 | 88.4 | 45 | 15.39 | 8.55 | 25 | 30 | 15 |
| **28** | 4.7175 | 79.56 | 45 | 13.68 | 5.13 | 30 | 15 | 13 |
| **29** | 4.995 | 44.2 | 46 | 15.39 | 6.84 | 40 | 34 | 25 |
| **30** | 5.3835 | 79.56 | 40 | 13.68 | 5.13 | 25 | 32 | 30 |
| **31** | 4.995 | 70.72 | 45 | 15.39 | 10.26 | 35 | 25 | 20 |
| **32** | 4.7175 | 79.56 | 43.5 | 10.26 | 3.42 | 25 | 20 | 16 |
| **33** | 4.44 | 44.2 | 40 | 13.68 | 3.42 | 23 | 13 | 12 |
| **34** | 4.995 | 88.4 | 45 | 10.26 | 3.42 | 35 | 25 | 26 |
| **35** | 4.7175 | 61.88 | 40 | 13.68 | 5.13 | 20 | 30 | 25 |
| **36** | 4.1625 | 44.2 | 40 | 15.39 | 3.42 | 35 | 25 | 20 |
| **37** | 4.995 | 79.56 | 45 | 13.68 | 6.84 | 25 | 30 | 23 |
| **38** | 5.2725 | 61.88 | 40 | 11.97 | 5.13 | 24 | 15 | 13 |
| **39** | 5.2725 | 79.56 | 40 | 10.26 | 1.71 | 35 | 25 | 24 |
| **40** | 4.7175 | 79.56 | 45 | 8.55 | 5.13 | 15 | 18 | 11 |
| **median** | 4.995 | 75.14 | 45 | 10.26 | 3.42 | 23 | 22 | 19.5 |
| **Refrence range** | (4.16 - 6.38 mmol/L | F:44.2-79.56 | 35-52 g/l | To17.1umol/l | To3.42umol/l | To 38 U/L | F: to 35  M:to 55 | F:to 35  M:to 55 |

Table І c: continued clinical finding of control group:

| **No** | **HBs AG** | **HCV Igg** | **PCR** | **α FP(ng/ml)** | **5-mc%** |
| --- | --- | --- | --- | --- | --- |
| **1** | negative | negative | negative | 3.3 | 1.34 |
| **2** | negative | negative | negative | 2.25 | 1.23 |
| **3** | negative | negative | negative | 5.23 | 0.53 |
| **4** | negative | negative | negative | 3.33 | 4.17 |
| **5** | negative | negative | negative | 6.28 | 0.28 |
| **6** | negative | negative | negative | 3.14 | 2.78 |
| **7** | negative | negative | negative | 2.18 | 5.44 |
| **8** | negative | negative | negative | 3.98 | 1.01 |
| **9** | negative | negative | negative | 4.32 | 11.89 |
| **10** | negative | negative | negative | 2.13 | 8.84 |
| **11** | negative | negative | negative | 3.89 | 0.64 |
| **12** | negative | negative | negative | 2.34 | 1.69 |
| **13** | negative | negative | negative | 5.5 | 4.04 |
| **14** | negative | negative | negative | 5.5 | 2.23 |
| **15** | negative | negative | negative | 2.3 | 2.32 |
| **16** | negative | negative | negative | 5.4 | 0.50 |
| **17** | negative | negative | negative | 4.34 | 3.74 |
| **18** | negative | negative | negative | 4.98 | 0.50 |
| **19** | negative | negative | negative | 6.43 | 0.75 |
| **20** | negative | negative | negative | 5.28 | 0.08 |
| **21** | negative | negative | negative | 4.33 | 29.36 |
| **22** | negative | negative | negative | 5.33 | 4.05 |
| **23** | negative | negative | negative | 1.34 | 11.15 |
| **24** | negative | negative | negative | 5.33 | 3.66 |
| **25** | negative | negative | negative | 5.33 | 4.60 |
| **26** | negative | negative | negative | 2.23 | 9.61 |
| **27** | negative | negative | negative | 5.23 | 9.44 |
| **28** | negative | negative | negative | 3.33 | 2.69 |
| **29** | negative | negative | negative | 2.23 | 1.50 |
| **30** | negative | negative | negative | 5.3 | 1.95 |
| **31** | negative | negative | negative | 3.12 | 1.23 |
| **32** | negative | negative | negative | 4.25 | 0.80 |
| **33** | negative | negative | negative | 4.56 | 3.79 |
| **34** | negative | negative | negative | 3.67 | 2.94 |
| **35** | negative | negative | negative | 5.23 | 1.75 |
| **36** | negative | negative | negative | 4.87 | 5.70 |
| **37** | negative | negative | negative | 3.33 | 1.50 |
| **38** | negative | negative | negative | 4.45 | 1.78 |
| **39** | negative | negative | negative | 5.24 | 3.58 |
| **40** | negative | negative | negative | 3.87 | 8.37 |
| **median** |  |  |  | 4.32 | 2.5 |
| **Min-Max** |  |  |  | 1.34-6.4 | 0.079-29.36 |
| **Refrence range** |  |  |  | Up to 8.5ng/ml |  |

Table ІІ a: clinical finding in patient group (HCV) ІІa:

| **No** | **Age (years)** | **sex** | **Child class** | **Hb(g/dl)** | **WBCs x 1000** | **Plts x1000** | **PT(sec)** | **INR** |
| --- | --- | --- | --- | --- | --- | --- | --- | --- |
| **1** | 45 | F | B | 12 | 3.93 | 130 | 16.3 | 1.8 |
| **2** | 50 | F | A | 13 | 3.01 | 297 | 12.5 | 1.1 |
| **3** | 72 | F | C | 10.4 | 5.66 | 182 | 14.6 | 1.5 |
| **4** | 58 | F | B | 9.9 | 12.2 | 347 | 15 | 1.6 |
| **5** | 40 | M | C | 7.5 | 4.43 | 55 | 17.6 | 2.1 |
| **6** | 40 | F | A | 13 | 4 | 270 | 13 | 1.2 |
| **7** | 60 | M | C | 11.6 | 3.57 | 84 | 17.4 | 2.1 |
| **8** | 50 | M | C | 7.1 | 5.08 | 50 | 16.2 | 1.8 |
| **9** | 69 | F | B | 9 | 10.5 | 64 | 13.6 | 1.3 |
| **10** | 50 | F | C | 10.2 | 5.22 | 170 | 12.5 | 1.07 |
| **11** | 45 | F | C | 9.6 | 14.2 | 205 | 25 | 4.4 |
| **12** | 46 | F | B | 11.1 | 6.65 | 112 | 18.1 | 2.2 |
| **13** | 60 | M | A | 10 | 5 | 100 | 27 | 4.8 |
| **14** | 67 | F | B | 10.7 | 3.49 | 200 | 14.4 | 1.4 |
| **15** | 45 | F | C | 11 | 5 | 200 | 12.5 | 1.1 |
| **16** | 45 | F | C | 12.8 | 7.15 | 120 | 16 | 1.8 |
| **17** | 66 | F | C | 12 | 5 | 200 | 15 | 1.6 |
| **18** | 55 | F | B | 10.1 | 6.29 | 160 | 14.5 | 1.5 |
| **19** | 54 | F | B | 11.1 | 6.65 | 112 | 18 | 2.2 |
| **20** | 60 | M | C | 8.2 | 4.5 | 46 | 22.1 | 3.3 |
| **21** | 54 | M | B | 11.5 | 15 | 200 | 12.5 | 1.1 |
| **22** | 55 | M | B | 11 | 5 | 300 | 14 | 1.4 |
| **23** | 64 | M | C | 8.2 | 13.8 | 42 | 26 | 4.5 |
| **24** | 45 | M | C | 10.2 | 3.77 | 100 | 16 | 1.8 |
| **25** | 45 | M | B | 9.4 | 3.8 | 70 | 15.3 | 1.6 |
| **26** | 64 | M | B | 8.7 | 2.74 | 45 | 16.6 | 1.9 |
| **27** | 60 | M | B | 9 | 6.65 | 112 | 16.6 | 1.9 |
| **28** | 56 | F | A | 11 | 6 | 200 | 27 | 4.8 |
| **29** | 55 | F | B | 12.5 | 4.9 | 250 | 15.4 | 1.6 |
| **30** | 50 | M | A | 14 | 9.65 | 147 | 13.7 | 1.3 |
| **31** | 49 | F | A | 15.4 | 10.56 | 254 | 13 | 1.2 |
| **32** | 50 | M | A | 14 | 10.4 | 80 | 13 | 1.2 |
| **33** | 50 | M | C | 9 | 7.06 | 80 | 17 | 2 |
| **34** | 70 | M | C | 15 | 10 | 300 | 21.6 | 3.1 |
| **35** | 65 | M | A | 15 | 7 | 250 | 13 | 1.3 |
| **36** | 50 | F | B | 11 | 6 | 170 | 12.5 | 1.1 |
| **37** | 57 | F | B | 12 | 8 | 200 | 12.5 | 1.1 |
| **38** | 54 | M | C | 11.1 | 4.5 | 78 | 21.6 | 3.1 |
| **39** | 40 | M | A | 14 | 4 | 250 | 13 | 1.2 |
| **40** | 64 | M | A | 13 | 4 | 150 | 14 | 1.3 |
| **41** | 67 | M | B | 9.9 | 5.24 | 55 | 13.6 | 1.3 |
| **42** | 60 | F | B | 9.4 | 4 | 100 | 13 | 1.2 |
| **43** | 67 | F | C | 9 | 5 | 100 | 13 | 1.2 |
| **44** | 60 | F | B | 7 | 3 | 56 | 12.5 | 1.1 |
| **45** | 55 | F | A | 11 | 4.5 | 160 | 12.5 | 1.1 |
| **46** | 60 | M | A | 14 | 6 | 250 | 12.5 | 1.1 |
| **47** | 58 | M | B | 12 | 9 | 100 | 15 | 1.6 |
| **48** | 46 | M | B | 9.2 | 4.25 | 45 | 15 | 1.6 |
| **49** | 67 | F | A | 13 | 5 | 200 | 15 | 1.6 |
| **50** | 66 | M | A | 12 | 5 | 150 | 16 | 1.6 |
| **51** | 56 | F | A | 12 | 5 | 200 | 12.5 | 1.1 |
| **52** | 55 | M | C | 8 | 9.2 | 55 | 25 | 4.2 |
| **53** | 38 | M | C | 8.3 | 12 | 92 | 24 | 3.9 |
| **54** | 37 | F | B | 8.1 | 3.8 | 206 | 14 | 1.3 |
| **55** | 40 | M | B | 13 | 5 | 200 | 12.5 | 1.1 |
| **56** | 55 | M | A | 13 | 6 | 150 | 13.6 | 1.3 |
| **57** | 62 | M | B | 9.5 | 8 | 150 | 13 | 1.2 |
| **58** | 50 | M | B | 10 | 9 | 200 | 12.5 | 1.1 |
| **59** | 50 | M | B | 12 | 10 | 150 | 13.6 | 1.3 |
| **60** | 68 | F | B | 12 | 8 | 140 | 15.1 | 1.6 |
| **Median** | **55** |  |  | **11** | **5** | **150** | **14.5** | **1.5** |
| **Min-max** | **37-72** |  |  | **7-15** | **3-15** | **42-347** | **12.5-27** | **1.07-4.8** |
| **Reference range** |  |  |  | F:11.7-15.5  M:12.5-17.5 | 4-11 | 150-450 | 11.5-14 | To 1.3 |

Table ІІ b: continued clinical finding in patient group HCV ІІa:

| **No** | **Glucose**  **(mmol/l)** | **Creatinine**  **(umol/l)** | **Albumin**  **(g/l)** | **T.bil**  **(umol/l)** | **D.bil**  **(umol/l)** | **GGT**  **(U/L)** | **AST**  **(U/L)** | **ALT**  **(U/L)** |
| --- | --- | --- | --- | --- | --- | --- | --- | --- |
| **1** | 5.55 | 77.792 | 19 | 32.49 | 20.52 | 26.8 | 90 | 46 |
| **2** | 5.106 | 79.56 | 44 | 6.84 | 1.71 | 13 | 20 | 12 |
| **3** | 9.99 | 53.04 | 25 | 75.24 | 39.33 | 69 | 50 | 30 |
| **4** | 8.88 | 123.76 | 22 | 6.84 | 1.71 | 41 | 64 | 29 |
| **5** | 5.55 | 97.24 | 22 | 41.04 | 25.65 | 26 | 66 | 47 |
| **6** | 11.1 | 88.4 | 38.5 | 6.84 | 1.71 | 57 | 154 | 235 |
| **7** | 5.9385 | 106.08 | 24 | 114.57 | 59.85 | 28 | 56 | 24 |
| **8** | 5.2725 | 238.68 | 27 | 56.43 | 17.1 | 28 | 39 | 40 |
| **9** | 7.3815 | 88.4 | 24 | 25.65 | 10.26 | 36 | 60 | 42 |
| **10** | 6.105 | 176.8 | 24 | 15.39 | 8.55 | 59 | 110 | 208 |
| **11** | 6.327 | 79.56 | 32 | 22.23 | 11.97 | 38 | 64 | 37 |
| **12** | 12.765 | 79.56 | 19 | 37.62 | 18.81 | 18 | 47 | 24 |
| **13** | 8.9355 | 44.2 | 30 | 11.97 | 5.13 | 35 | 30 | 19 |
| **14** | 4.551 | 70.72 | 24 | 34.2 | 20.52 | 50 | 169 | 52 |
| **15** | 4.8285 | 70.72 | 28 | 78.66 | 46.17 | 60 | 68 | 18 |
| **16** | 4.995 | 79.56 | 22 | 53.01 | 30.78 | 40 | 39 | 71 |
| **17** | 4.7175 | 70.72 | 21 | 17.1 | 8.55 | 45 | 111 | 66 |
| **18** | 5.55 | 79.56 | 23 | 20.52 | 11.97 | 50 | 37 | 21 |
| **19** | 5.2725 | 79.56 | 27 | 25.65 | 11.97 | 55 | 56 | 28 |
| **20** | 8.325 | 79.56 | 24 | 82.08 | 39.33 | 35 | 32 | 15 |
| **21** | 12.765 | 176.8 | 24 | 11.97 | 5.13 | 50 | 42 | 22 |
| **22** | 9.99 | 141.44 | 25 | 20.52 | 13.68 | 45 | 85 | 49 |
| **23** | 6.105 | 132.6 | 19 | 271.89 | 198.36 | 55 | 25 | 29 |
| **24** | 5.2725 | 141.44 | 18 | 41.04 | 22.23 | 16 | 32 | 19 |
| **25** | 12.987 | 79.56 | 30 | 19.836 | 11.97 | 33 | 24 | 13 |
| **26** | 11.1 | 79.56 | 22 | 20.52 | 11.97 | 17 | 69 | 48 |
| **27** | 8.88 | 79.56 | 22 | 30.78 | 13.68 | 40 | 47 | 16 |
| **28** | 4.8285 | 70.72 | 32 | 17.1 | 6.84 | 15 | 12 | 7 |
| **29** | 6.66 | 70.72 | 26 | 13.68 | 5.13 | 33 | 58 | 27 |
| **30** | 5.2725 | 79.56 | 21 | 6.84 | 1.71 | 11 | 12 | 11 |
| **31** | 3.441 | 70.72 | 22 | 3.42 | 1.71 | 10 | 38 | 17 |
| **32** | 2.8305 | 79.56 | 33 | 29.07 | 8.55 | 34 | 47 | 30 |
| **33** | 5.55 | 106.08 | 27 | 37.62 | 17.1 | 16 | 37 | 17 |
| **34** | 5.328 | 106.08 | 27 | 66.69 | 34.2 | 70 | 52 | 25 |
| **35** | 4.995 | 70.72 | 39 | 8.55 | 1.71 | 15 | 50 | 45 |
| **36** | 6.549 | 70.72 | 25 | 10.26 | 6.84 | 31 | 23 | 14 |
| **37** | 10.1565 | 106.08 | 15 | 15.39 | 5.13 | 42 | 71 | 46 |
| **38** | 5.55 | 88.4 | 21 | 164.16 | 97.47 | 24 | 86 | 39 |
| **39** | 7.215 | 79.56 | 40 | 8.55 | 3.42 | 25 | 100 | 130 |
| **40** | 10.545 | 79.56 | 40 | 42.75 | 17.1 | 15 | 50 | 10 |
| **41** | 9.546 | 88.4 | 24 | 23.94 | 10.26 | 73 | 48 | 21 |
| **42** | 11.8215 | 106.08 | 27 | 17.1 | 8.55 | 51 | 39 | 15 |
| **43** | 4.44 | 327.08 | 20 | 104.31 | 78.66 | 27 | 500 | 400 |
| **44** | 6.216 | 61.88 | 25 | 27.36 | 18.81 | 18 | 71 | 20 |
| **45** | 6.66 | 79.56 | 36 | 11.97 | 1.71 | 70 | 75 | 35 |
| **46** | 4.44 | 79.56 | 40 | 8.55 | 3.42 | 35 | 25 | 28 |
| **47** | 5.661 | 88.4 | 27 | 37.62 | 20.52 | 26 | 81 | 31 |
| **48** | 15.096 | 238.68 | 25 | 11.97 | 3.42 | 42 | 22 | 15 |
| **49** | 4.9395 | 79.56 | 39 | 17.1 | 11.97 | 35 | 59 | 36 |
| **50** | 8.325 | 79.56 | 21 | 18.81 | 3.42 | 39 | 110 | 85 |
| **51** | 4.995 | 79.56 | 40 | 8.55 | 3.42 | 15 | 17 | 10 |
| **52** | 5.328 | 70.72 | 21 | 112.86 | 94.05 | 35 | 40 | 15 |
| **53** | 5.6055 | 97.24 | 19 | 47.88 | 35.91 | 14 | 35 | 15 |
| **54** | 6.6045 | 97.24 | 24 | 8.55 | 3.42 | 16 | 30 | 17 |
| **55** | 4.7175 | 97.24 | 21 | 27.36 | 18.81 | 25 | 40 | 31 |
| **56** | 9.879 | 88.4 | 36 | 6.84 | 1.71 | 44 | 63 | 64 |
| **57** | 5.217 | 79.56 | 26 | 30.78 | 13.68 | 50 | 68 | 24 |
| **58** | 5.55 | 97.24 | 25 | 27.36 | 15.39 | 164 | 105 | 79 |
| **59** | 4.995 | 88.4 | 27 | 25.65 | 11.97 | 40 | 35 | 30 |
| **60** | 5.55 | 79.56 | 28 | 30.78 | 15.39 | 35 | 40 | 35 |
| **Median** | 5.63325 | 79.56 | 25 | 24.795 | 11.97 | **35** | **50** | **28.5** |
| **Refrence range** | (4.16 - 6.38 mmol/L | F:44.2-79.56 | 35-52 g/l | To17.1umol/l | To3.42umol/l | To 38 U/L | F: to 35  M:to 55 | F:to 35  M:to 55 |

Table ІІ c: continued lab finding in patient group HCV ІІ a:

| **No** | **HBs Ag** | **HCV Igg** | **PCR** | **Afp(ng/ml)** | **5-mc %** |
| --- | --- | --- | --- | --- | --- |
| **1** | negative | positive | 560000 | 5.02 | 1.86 |
| **2** | negative | positive | 2500 | 2.18 | 1.02 |
| **3** | negative | positive | 35000 | 1.8 | 0.38 |
| **4** | negative | positive | 5000000 | 4.05 | 1.52 |
| **5** | negative | positive | 340000 | 1.74 | 1.86 |
| **6** | negative | positive | 800000 | 5.34 | 0.08 |
| **7** | negative | positive | 10000 | 2.89 | 0.20 |
| **8** | negative | positive | 5600 | 3.24 | 0.89 |
| **9** | negative | positive | 70000 | 2.66 | 3.20 |
| **10** | negative | positive | 1500 | 20 | 1.19 |
| **11** | negative | positive | 2600 | 10.3 | 3.55 |
| **12** | negative | positive | 5000000 | 3.39 | 1.20 |
| **13** | negative | positive | 5800 | 2 | 1.99 |
| **14** | negative | positive | 20000 | 12.6 | 1.22 |
| **15** | negative | positive | 650000 | 10 | 4.01 |
| **16** | negative | positive | 350000 | 2.19 | 2.86 |
| **17** | negative | positive | 90000 | 50 | 3.63 |
| **18** | negative | positive | 6000000 | 8.8 | 2.64 |
| **19** | negative | positive | 550000 | 12.3 | 8 |
| **20** | negative | positive | 350000 | 12 | 2.02 |
| **21** | negative | positive | 65000 | 4.9 | 3.81 |
| **22** | negative | positive | 46000 | 5.5 | 12.13 |
| **23** | negative | positive | 350000 | 8.8 | 3.03 |
| **24** | negative | positive | 7500000 | 10 | 5.63 |
| **25** | negative | positive | 4000 | 6.2 | 22.0 |
| **26** | negative | positive | 280000 | 4 | 10.90 |
| **27** | negative | positive | 550000 | 10 | 0.31 |
| **28** | negative | positive | 350000 | 9.3 | 3.84 |
| **29** | negative | positive | 850000 | 11.3 | 0.26 |
| **30** | negative | positive | 80000 | 3.3 | 0.53 |
| **31** | negative | positive | 6000000 | 4.5 | 2.01 |
| **32** | negative | positive | 8500 | 4 | 3.86 |
| **33** | negative | positive | 64000 | 11 | 0.19 |
| **34** | negative | positive | 5000 | 5 | 0.98 |
| **35** | negative | positive | 4200 | 8.12 | 17.0 |
| **36** | negative | positive | 250000 | 6.32 | 0.18 |
| **37** | negative | positive | 50000 | 7.33 | 2.60 |
| **38** | negative | positive | 640000 | 10 | 1.06 |
| **39** | negative | positive | 5000 | 5.15 | 4.42 |
| **40** | negative | positive | 3000 | 6.23 | 7.22 |
| **41** | negative | positive | 15000 | 5.22 | 2.93 |
| **42** | negative | positive | 4000 | 10 | 5.12 |
| **43** | negative | positive | 65000 | 12.4 | 3.43 |
| **44** | negative | positive | 35000 | 7.3 | 2.39 |
| **45** | negative | positive | 4200 | 8 | 4.27 |
| **46** | negative | positive | 8000 | 6.6 | 8.06 |
| **47** | negative | positive | 20000 | 7.15 | 0.13 |
| **48** | negative | positive | 6000000 | 6.14 | 0.43 |
| **49** | negative | positive | 25000 | 8.8 | 1.51 |
| **50** | negative | positive | 3500 | 11.3 | 6.03 |
| **51** | negative | positive | 450000 | 6.2 | 0.84 |
| **52** | negative | positive | 25000 | 10.26 | 2.50 |
| **53** | negative | positive | 580000 | 9 | 4.16 |
| **54** | negative | positive | 25000 | 10 | 0.45 |
| **55** | negative | positive | 65000 | 9.14 | 2.31 |
| **56** | negative | positive | 58000 | 10 | 8.60 |
| **57** | negative | positive | 8000 | 12.4 | 1.13 |
| **58** | negative | positive | 500000 | 9.3 | 8.30 |
| **59** | negative | positive | 25000 | 10 | 0.69 |
| **60** | negative | positive | 65000 | 17.2 | 4.26 |
| **Median** |  |  | **64500** | **7.665** | **2.45** |
| **Min-Max** |  |  | **1500-7500000** | **1.74-50** | **0.08-22** |
| **Refrence range** |  |  |  | **Up to 8.5 ng/ml** |  |

Table ІІІ a: lab finding in patient group (HCC):ІІb

| **No** | **Age**  **(years)** | **Sex** | **Child score** | **Hb(g/dl)** | **WBCs**  **X1000** | **Plts**  **X1000** | **PT(sec)** | **INR** |
| --- | --- | --- | --- | --- | --- | --- | --- | --- |
| **1** | 63 | M | B | 8 | 3.3 | 40 | 14.2 | 1.4 |
| **2** | 50 | M | C | 16.1 | 23.14 | 161 | 23.9 | 3.8 |
| **3** | 60 | M | B | 11.2 | 5.17 | 90 | 16.7 | 1.9 |
| **4** | 60 | M | C | 10.5 | 7.56 | 113 | 16.4 | 1.9 |
| **5** | 50 | F | C | 9.7 | 8.93 | 97 | 13.1 | 1.2 |
| **6** | 50 | F | B | 9.2 | 4.6 | 127 | 12.5 | 1.1 |
| **7** | 54 | M | C | 10.5 | 6.98 | 112 | 14.8 | 1.5 |
| **8** | 50 | M | C | 9.9 | 8.9 | 41 | 14.6 | 1.5 |
| **9** | 55 | M | B | 11 | 6 | 90 | 15 | 1.27 |
| **10** | 60 | M | C | 10.2 | 4.74 | 95 | 14.9 | 1.6 |
| **11** | 60 | M | C | 6.7 | 3.28 | 40 | 15.3 | 1.6 |
| **12** | 50 | M | C | 10.4 | 9 | 100 | 16.1 | 1.8 |
| **13** | 50 | M | B | 11.9 | 4 | 38 | 15.3 | 1.6 |
| **14** | 69 | F | C | 10 | 7 | 113 | 15 | 1.6 |
| **15** | 73 | F | B | 11.5 | 15.3 | 100 | 13.1 | 1.2 |
| **16** | 59 | M | C | 9.1 | 11.1 | 174 | 19.2 | 2.5 |
| **17** | 50 | M | C | 7.7 | 8.21 | 218 | 20.1 | 2.7 |
| **18** | 61 | F | B | 9 | 10 | 100 | 12.5 | 1.1 |
| **19** | 60 | F | C | 8.5 | 16.68 | 64 | 27.9 | 4.8 |
| **20** | 36 | F | B | 10 | 15 | 151 | 20.7 | 2.9 |
| **Median** | **57** |  |  | **10** | **7.88** | **100** | **15** | **1.6** |
| **Min-Max** | **36-73** |  |  | **6.7-16.1** | **3-23** | **38-218** | **12.5-27.9** | **1.1-4.8** |
| **Reference range** |  |  |  | F:11.7-15.5  M:12.5-17.5 | 4-11 | 150-450 | 11.5-14 | To 1.3 |

**Table** ІІІ b: continued lab finding in patient group HCC: ІІb

| **No** | **Glucose**  **(mmol/l)** | **Creatinine**  **(umol/l)** | **Albumin**  **(g/l)** | **T.bil**  **(umol/l)** | **D.bil**  **(umol/l)** | **GGT**  **(U/L)** | **AST**  **(U/L)** | **ALT**  **(U/L|)** |
| --- | --- | --- | --- | --- | --- | --- | --- | --- |
| **1** | 13.32 | 97.24 | 23 | 29.07 | 18.81 | 188 | 135 | 115 |
| **2** | 10.1565 | 185.64 | 21 | 44.46 | 27.36 | 61.6 | 149 | 46 |
| **3** | 9.435 | 106.08 | 27 | 30.78 | 13.68 | 33 | 68 | 30 |
| **4** | 6.105 | 106.08 | 20 | 196.65 | 157.32 | 71 | 167 | 85 |
| **5** | 13.209 | 79.56 | 26 | 41.04 | 27.36 | 400 | 218 | 64 |
| **6** | 4.995 | 88.4 | 29 | 11.97 | 6.84 | 33 | 24 | 8 |
| **7** | 4.44 | 106.08 | 17 | 64.98 | 41.04 | 86 | 112 | 38 |
| **8** | 4.7175 | 88.4 | 22 | 54.72 | 37.62 | 192 | 77 | 27 |
| **9** | 6.1605 | 79.56 | 31 | 88.92 | 68.4 | 155 | 274 | 111 |
| **10** | 9.99 | 70.72 | 20 | 242.82 | 193.23 | 88 | 99 | 15 |
| **11** | 10.545 | 495.04 | 20 | 15.39 | 11.97 | 70 | 16 | 18 |
| **12** | 5.661 | 106.08 | 24 | 83.79 | 63.27 | 80 | 572 | 412 |
| **13** | 6.2715 | 88.4 | 32 | 36.594 | 10.26 | 70 | 51 | 24 |
| **14** | 4.9395 | 123.76 | 28 | 174.42 | 128.25 | 119 | 638 | 56 |
| **15** | 6.105 | 70.72 | 26 | 35.91 | 30.78 | 305 | 185 | 147 |
| **16** | 5.106 | 70.72 | 14 | 208.62 | 136.8 | 57 | 118 | 52 |
| **17** | 6.771 | 141.44 | 23 | 336.87 | 253.08 | 61 | 130 | 34 |
| **18** | 5.2725 | 70.72 | 28 | 11.97 | 1.71 | 44 | 52 | 45 |
| **19** | 6.3825 | 79.56 | 19 | 232.56 | 143.64 | 26 | 39 | 16 |
| **20** | 8.9355 | 88.4 | 24 | 41.04 | 25.65 | 49 | 50 | 40 |
| **MEDIAN** | 6.216 | 88.4 | 23.5 | 49.59 | 34.2 | **70.5** | **115** | **42.5** |
| **Reference range** | (4.16 - 6.38 mmol/L | F:44.2-79.56 | 35-52 g/l | To17.1umol/l | To3.42umol/l | To 38 U/L | F: to 35  M:to 55 | F:to 35  M:to 55 |

Table ІІІ c: continued lab finding of patient group HCC ІІb:

| **No** | **HBs Ag** | **HCV Igg** | **PCR** | **AFP(ng/ml)** | **5-mc%** | **BCLC** |
| --- | --- | --- | --- | --- | --- | --- |
| **1** | negative | positive | 7000 | 233 | 1.66 | B |
| **2** | negative | positive | 2500 | 367 | 2.11 | B |
| **3** | negative | positive | 3000000 | 5 | 2.39 | C |
| **4** | negative | positive | 8000 | 250 | 2.40 | B |
| **5** | negative | positive | 750000 | 75 | 0.52 | C |
| **6** | negative | positive | 680000 | 200 | 7.80 | C |
| **7** | negative | positive | 2000000 | 150 | 1.58 | C |
| **8** | negative | positive | 550000 | 300 | 7.14 | D |
| **9** | negative | positive | 2500 | 150 | 1.22 | B |
| **10** | negative | positive | 632000 | 111.3 | 0.19 | D |
| **11** | negative | positive | 150000 | 264 | 0.88 | C |
| **12** | negative | positive | 480000 | 50 | 4.39 | C |
| **13** | negative | positive | 658000 | 48 | 3.82 | D |
| **14** | negative | positive | 310000 | 500 | 0.38 | D |
| **15** | negative | positive | 9000000 | 341 | 0.18 | C |
| **16** | negative | positive | 580000 | 294 | 6.44 | C |
| **17** | negative | positive | 258000 | 121 | 5.51 | D |
| **18** | negative | positive | 3000 | 50.5 | 0.16 | B |
| **19** | negative | positive | 56000 | 436 | 8.87 | C |
| **20** | negative | positive | 205000 | 40.4 | 5.09 | C |
| **Median** |  |  | **395000** | **175** | **2.25** | **C** |
| **Min-Max** |  |  | **2500-9000000** | **5-500** | **0.16-8.86** |  |
| **Reference range** |  |  |  | **Up to 8.5 ng/ml** |  |  |

**Supplementary molecular data for individual case**

**Table І: Molecular**  finding of control group:

| **No** | **5-mc%** |
| --- | --- |
| **1** | 1.34 |
| **2** | 1.23 |
| **3** | 0.53 |
| **4** | 4.17 |
| **5** | 0.28 |
| **6** | 2.78 |
| **7** | 5.44 |
| **8** | 1.01 |
| **9** | 11.89 |
| **10** | 8.84 |
| **11** | 0.64 |
| **12** | 1.69 |
| **13** | 4.04 |
| **14** | 2.23 |
| **15** | 2.32 |
| **16** | 0.50 |
| **17** | 3.74 |
| **18** | 0.50 |
| **19** | 0.75 |
| **20** | 0.08 |
| **21** | 29.36 |
| **22** | 4.05 |
| **23** | 11.15 |
| **24** | 3.66 |
| **25** | 4.60 |
| **26** | 9.61 |
| **27** | 9.44 |
| **28** | 2.69 |
| **29** | 1.50 |
| **30** | 1.95 |
| **31** | 1.23 |
| **32** | 0.80 |
| **33** | 3.79 |
| **34** | 2.94 |
| **35** | 1.75 |
| **36** | 5.70 |
| **37** | 1.50 |
| **38** | 1.78 |
| **39** | 3.58 |
| **40** | 8.37 |
| **median** | 2.5 |
| **Min-Max** | 0.079-29.36 |
| **Refrence range** |  |

**Table ІІ : Molecualr**  finding in patient group HCV ІІ a:

| **No** | **5-mc %** |
| --- | --- |
| **1** | 1.86 |
| **2** | 1.02 |
| **3** | 0.38 |
| **4** | 1.52 |
| **5** | 1.86 |
| **6** | 0.08 |
| **7** | 0.20 |
| **8** | 0.89 |
| **9** | 3.20 |
| **10** | 1.19 |
| **11** | 3.55 |
| **12** | 1.20 |
| **13** | 1.99 |
| **14** | 1.22 |
| **15** | 4.01 |
| **16** | 2.86 |
| **17** | 3.63 |
| **18** | 2.64 |
| **19** | 8 |
| **20** | 2.02 |
| **21** | 3.81 |
| **22** | 12.13 |
| **23** | 3.03 |
| **24** | 5.63 |
| **25** | 22.0 |
| **26** | 10.90 |
| **27** | 0.31 |
| **28** | 3.84 |
| **29** | 0.26 |
| **30** | 0.53 |
| **31** | 2.01 |
| **32** | 3.86 |
| **33** | 0.19 |
| **34** | 0.98 |
| **35** | 17.0 |
| **36** | 0.18 |
| **37** | 2.60 |
| **38** | 1.06 |
| **39** | 4.42 |
| **40** | 7.22 |
| **41** | 2.93 |
| **42** | 5.12 |
| **43** | 3.43 |
| **44** | 2.39 |
| **45** | 4.27 |
| **46** | 8.06 |
| **47** | 0.13 |
| **48** | 0.43 |
| **49** | 1.51 |
| **50** | 6.03 |
| **51** | 0.84 |
| **52** | 2.50 |
| **53** | 4.16 |
| **54** | 0.45 |
| **55** | 2.31 |
| **56** | 8.60 |
| **57** | 1.13 |
| **58** | 8.30 |
| **59** | 0.69 |
| **60** | 4.26 |
| **Median** | **2.45** |
| **Min-Max** | **0.08-22** |
| **Refrence range** |  |

**Table ІІІ : Molecular** finding of patient group HCC ІІb:

| **No** | **5-mc%** |
| --- | --- |
| **1** | 1.66 |
| **2** | 2.11 |
| **3** | 2.39 |
| **4** | 2.40 |
| **5** | 0.52 |
| **6** | 7.80 |
| **7** | 1.58 |
| **8** | 7.14 |
| **9** | 1.22 |
| **10** | 0.19 |
| **11** | 0.88 |
| **12** | 4.39 |
| **13** | 3.82 |
| **14** | 0.38 |
| **15** | 0.18 |
| **16** | 6.44 |
| **17** | 5.51 |
| **18** | 0.16 |
| **19** | 8.87 |
| **20** | 5.09 |
| **Median** | **2.25** |
| **Min-Max** | **0.16-8.86** |
| **Reference range** |  |
